# Effect of *in vitro* growth on mouse oocyte competency, mitochondria and transcriptome

**DOI:** 10.1530/REP-21-0209

**Published:** 2021-08-12

**Authors:** Tomoya Takashima, Tsubasa Fujimaru, Yayoi Obata

**Affiliations:** 1 Department of Bioscience, Tokyo University of Agriculture, Tokyo, Japan

## Abstract

*In vitro* generation of fertile oocytes has been reported in several mammalian species. However, oocyte integrity is compromised by *in vitro* culture. Here, we aimed to understand the factors affecting oocyte competency by evaluating mitochondrial function and transcriptome as well as lipid metabolism in *in vivo*-derived oocytes and *in vitro* grown and matured (IVGM) oocytes under atmospheric (20%) and physiological (7%) O_2_ concentration. We used single-cell RNA-sequencing as well as Gene Ontology and KEGG analyses to identify the molecular pathways affecting the developmental competence of oocytes. Oocytes grown under 20% O_2_ conditions showed a significant decrease in mitochondrial membrane potential, upregulation of ceramide synthesis pathway-associated genes, and high ceramide accumulation compared with oocytes grown under 7% O_2_ conditions and *in vivo*-grown oocytes. This suggests that excess ceramide level causes mitochondrial dysfunction and poor developmental ability of the oocytes. Mitochondrial DNA copy number was lower in IVGM oocytes irrespective of O_2_ concentration in culture, although there was no common abnormality in the expression of genes related to mitochondrial biosynthesis. In contrast, some oocytes produced under 7% O_2_ conditions showed gene expression profiles similar to those of *in vivo*-grown oocytes. In these oocytes, the expression of transcription factors, including *Nobox*, was restored. *Nobox* expression correlated with the expression of genes essential for oocyte development. Thus, *Nobox* may contribute to the establishment of oocyte competency before and after the growth phase. The comprehensive analysis of IVGM oocytes presented here provides a platform for elucidating the mechanism underlying functional oocyte production* in vivo*.

## Introduction

Gametes are the only cells capable of transmitting genetic and epigenetic information to their descendants after fertilization. Oocyte, the female gamete, is responsible for the transmission of organelles, such as mitochondria and endoplasmic reticula, and providing maternal factors necessary for embryonic development. At the initial stage of oogenesis, oocytes contain only a small amount of cytoplasm and mitochondria (Cao *et al.* 2007). These oocytes are functionally immature and lack meiotic and developmental competencies (Sorensen & Wassarman 1976, Eppig *et al.* 1994). Once oocytes enter the growth phase, transcription and translation of maternal factors and biosynthesis of organelles are drastically activated (Mtango *et al.* 2008). As a result, cytoplasmic volume increases by approximately 125-fold, and mitochondrial DNA (mtDNA) copy number increases by approximately 30-fold, as observed in mouse oocytes (Cao *et al.* 2007), leading to the acquisition of functional competency. However, the entire process of generating competent oocytes remains unknown.

Mitochondria perform multiple functions, including ATP production, Ca^2+^ homeostasis, steroid hormone biosynthesis, and apoptosis (Green & Reed 1998). During oocyte growth, the mitochondrial matrix becomes highly dense, indicating the accumulation of factors essential for ATP production and mitochondrial biosynthesis in the fully grown oocytes (Sathananthan & Trounson 2000). As ATP produced in oocytes is utilized for meiosis and early embryonic development (Yu *et al.* 2010, Dalton *et al.* 2014), ATP content and mitochondrial membrane potential are indexes of oocyte competency. In fact, aging, obesity, and diabetes reportedly result in impaired female fertility and are associated with mitochondrial dysfunction, including reduced mitochondrial membrane potential, low ATP content, and abnormal cellular localization, in the oocytes (Wang *et al.* 2009, Wang & Moley 2010, Vural *et al.* 2015, Wu *et al.* 2015, Li *et al.* 2018). High negative potential of the inner mitochondrial membrane, which is created by H^+^ pumps, is essential for the production of ATP and inhibition of cytochrome c release, that is, inhibition of apoptosis, and is an indicator of mitochondrial integrity.

Several studies have reported the production of fertile oocytes from immature ovarian follicles in mice, cows, and pigs. We and others have successfully produced functionally mature oocytes via *in vitro* growth (IVG) of mouse secondary follicles (Morohaku *et al.* 2016*a*,*b*, Mizumachi *et al.* 2018, Morohaku 2019). However, the competency of these IVG oocytes was markedly lower than that of *in vivo*-grown oocytes, and only a few of them developed to term after *in vitro* maturation (IVM), *in vitro* fertilization (IVF), and embryo transfer (ET). More recently, we evaluated the IVG conditions and demonstrated a significant increase in the yield of fertile oocytes by the addition of high molecular weight polyvinylpyrrolidone (PVP) to the medium and reducing the O_2_ concentration from 20 to 7% (Ota *et al.* 2021). These results were consistent with previous reports on optimal O_2_ concentration for IVG of bovine and murine oocytes (Eppig & Wigglesworth 1995, Hirao *et al.* 2012). Indeed, physiological O_2_ concentrations range from 10.5 to 1.4% in the reproductive tract (Fischer *et al.* 1992, de Castro *et al.* 2008, Redding *et al.* 2008). IVM oocytes are known to exhibit reduced developmental ability accompanied by decreased mitochondrial membrane potential and ATP content, increased reactive oxygen species (ROS), and abnormal mitochondrial localization (Nagai *et al.* 2006, Yuan *et al.* 2016, Hosseinzadeh Shirzeyli *et al.* 2020). In the somatic cell, O_2_ concentration affects the efficiency of ATP production by mitochondria, regulation of gene expression, and cell fate (Pfander *et al.* 2003, Kenneth & Rocha 2008). Therefore, mitochondria are pivotal in influencing the developmental competence of IVG oocytes. However, whether O_2_ concentration during IVG affects oocyte mitochondrial function and the mechanisms underlying reduced developmental competence of IVG oocytes remain to be elucidated. Identifying factors responsible for abnormalities in IVG oocytes will help understand the process of functional oocyte development *in vivo*. The present study aimed to determine the effect of O_2_ concentration in culture on mitochondrial quality and quantity in IVG oocytes. Furthermore, we aimed to identify the molecular pathway impacting mitochondrial function and developmental competence of IVG oocytes through single-cell RNA-sequencing (scRNA-seq) and lipid analyses.

## Materials and methods

### Animals

All animals were purchased from CLEA Japan Inc. B6D2F1 (C57BL/6N × DBA/2) mice were used for all experiments. The animals were maintained in accordance with the guidelines of the Science Council of Japan, and all experiments were approved by the Institutional Animal Care and Use Committee of the Tokyo University of Agriculture (approval number: 2020048).

### Culture

All culture experiments for oocyte and embryo production were conducted as described previously (Morohaku *et al.* 2017) (Supplementary materials and methods, see section on supplementary materials given at the end of this article).

### Oocyte isolation for analyses

Oocytes at the germinal vesicle (GV) stage were collected from explants at day 12 of IVG. As control, *in vivo*-grown GV oocytes were collected from Graafian follicles of adult mice 44–48 h after administration of 5 IU of equine chorionic gonadotropin (eCG; ASKA Pharmaceutical). If we needed to select a part of COCs to be subjected to two or more analyses, we numbered each COC and chose the number randomly without observation. Then, survived COCs were used for each analysis. To isolate IVG oocytes and *in vivo*-grown GV oocytes, all cumulus cells were removed by pipetting using fine pulled-glass capillary in M2 medium containing 240 µM dibutyryl cyclic AMP (Sigma–Aldrich).

IVGM oocytes at the MII stage were isolated from expanded COCs 17 h after IVM. To obtain control oocytes, 5 IU of hCG was administered to adult mice 44–48 h after 5 IU eCG administration; after 14–16 h, these *in vivo*-derived MII oocytes were collected. To isolate *in vivo*-derived MII oocytes and IVGM oocytes, all cumulus cells were removed by pipetting in M2 medium with or without 300 mIU of hyaluronidase (Sigma–Aldrich).

### Analysis of mitochondria

IVG/IVGM oocytes and *in vivo*-derived oocytes were simultaneously observed using the Nikon A1 confocal microscope (Nikon), and images were captured. Fluorescence intensity was measured with NIS-element software (Nikon).

To assess mitochondrial distribution, oocytes were observed after incubation with 100 nM MitoTracker DeepRed FM (Thermo Fisher) in M2 medium containing 1 µg/mL Hoechst 33342 (Dojindo) at 37°C for 1 h. The images which were acquired near the center of the oocytes in the Z-axis with GV in focus were subjected to the analysis. The circular regions of interest were drawn along the GV (perinuclear 30%) and oocyte (cortical 20%), and the oocyte was divided into two regions. Average intensities of perinuclear and cortical regions in each oocyte were measured. When the intensity ratio (perinuclear/cortical) was within the mean ratio ± 1 s.d. of* in vivo*-grown oocytes, mitochondrial distribution was judged as a normal pattern. When the ratio was without the mean ratio ± 1 s.d. of* in vivo*-grown oocytes, mitochondrial distribution was judged as an abnormal pattern.

To evaluate mitochondrial membrane potential, oocytes were incubated with 1% (v/v) JC-1 (Cayman Chemical) in M2 medium at 37°C for 15 min. After washing in M2 medium, the oocytes were observed. Mitochondrial membrane potential was evaluated by Intensities of J-aggregate (red fluorescence)/monomer (green fluorescence) in each oocyte.

ROS in oocytes were detected with CellROX Green (Thermo Fisher). Oocytes were observed after incubation with 5 µM CellROX Green in M2 medium at 37°C for 1 h.

Intercellular ATP content for each oocyte was measured with an Intracellular ATP assay kit ver.2 (Toyo Ink Group) using a SpectraMax i3 plate reader (Molecular Devices), following the manufacturer’s instructions. All samples were analyzed at least in duplicate.

For quantitative analysis of mtDNA copy numbers, zona pellucida of oocytes were digested with 0.5% (w/v) pronase (Sigma–Aldrich), and polar bodies were removed by pipetting. Individual oocytes were lysed using QIAamp DNA Micro Kit (QIAGEN), and mtDNA from each oocyte was eluted in 40 µL of water. Six microliters of this elution were subjected to quantitative PCR (q-PCR) in a 20-µL reaction containing specific primers (forward: 5′-AACCTGGCACTGAGTCACCA-3′, reverse: 5′-GGGTCTGAGTGTATATATCATGAAGAGAAT-3′), customized Taqman probe (FAM-TCTGTAGCCCTTTTTGTCACATGATC-TAMRA; Thermo Fisher), and TaqMan Universal Master Mix II (Thermo Fisher) (Cao *et al.* 2007). q-PCR was performed using QuantStudio 3 (Thermo Fisher) with the following conditions: 50°C for 2 min, 95°C for 10 min, and 40 cycles at 95°C for 15 s and 60°C for 60 s. The external standard used was a plasmid (pGEM -T Easy Vector Systems; Promega) in which the PCR product of mtDNA was cloned. All samples were analyzed in duplicate.

### scRNA-seq analysis of oocytes

Oocytes obtained via two independent culture experiments were subjected to scRNA-seq analysis. GV oocytes were treated with 0.5% (w/v) pronase in M2 medium to remove zona pellucida. Each oocyte was transferred into a microtube containing 7 µL PBS (−), and a cDNA library was generated using a QIAseq FX Single-Cell RNA Library kit (QIAGEN) following the manufacturer’s instructions. The GeneRead Size Selection kit (QIAGEN) was used to clean up the synthesized library. Quality assessment was performed using an Agilent DNA1000 kit (Agilent). Thereafter, quantity assessment was performed using a KAPA Library Quantification kit (KAPA). Finally, all libraries were mixed and subjected to single-end 50 bp sequencing using NextSeq 500 (Illumina).

Quality control of the sequences was performed using the Trim Galore! (v0.6.4) program with the following configurations: trimming the adapter sequence, minimal base quality, 25 and minimal read length after trimming, 35 nt (https://www.bioinformatics.babraham.ac.uk/projects/trim_galore/). scRNA-seq reads were aligned to the mouse genome assembly (mm10) using HISAT2 (v2.1.0) (Pertea *et al.* 2016). FeatureCounts (v1.6.5) program with Ensembl gene annotation (v81, mm10, 46073 genes) was used to estimate their abundances (Liao *et al.* 2014). The read densities (counts per million reads (CPM)) were converted to the log_2_ (CPM+1) scale (log_2_ expression level). Significant differentially expressed genes (DEGs) were identified using Wald test in DESeq2 (v1.20.1) with the adjusted *P*-value (padj) less than the 0.05 threshold among the groups (Love *et al.* 2014). Principal component analysis (PCA) and hierarchical clustering were performed with R (v3.5.1, https://www.R-project.org/) (Ihaka & Gentleman 1996). Gene Ontology (GO) (for Biological Process terms) and Kyoto Encyclopedia of Genes and Genomes (KEGG) pathway enrichment analyses were performed using DAVID (v6.8, *q*-value < 0.05) (http://david.abcc.ncifcrf.gov/) (Huang da *et al.* 2009). The common DEGs in 20%-IVG and 7%-IVG oocytes were classified into four groups with K-means clustering using R.

### Measurement of ceramide content

GV oocytes (100–200) after removal of cumulus cells were transferred into a microtube containing 20 µL of water. Pooled oocytes were frozen in liquid nitrogen and stored at –80°C until further analysis. Sample preparation and liquid chromatograph-mass spectrometry (LC-MS) analysis was performed at the Environmental Technology Department at the Chemicals Evaluation and Research Institute (CERI; Tokyo, Japan). To measure ceramide, lipid was extracted from the pooled sample by the chloroform-methanol extraction method. Lipid extract was analyzed by LC-MS (Nexera XR, Shimadzu; QTRAP 5500, AB sciex). An L-column2 C8 (2.0 × 50 mm) with 3 µm particles was used with 0.3 mL/min flow rate and 3 µL injection volume. The detected peak area in each sample was corrected using the internal standard ceramide (d18:1/16:0; Merck).

### Statistical analysis

Data were obtained from three or more independent culture experiments. Data are presented as mean ± s.d. for each group. Tukey–Kramer test was used for multiple comparisons among experimental groups to assess mitochondrial function and quantity. Chi-square test was used to assess the developmental ability of embryos produced from IVGM oocytes. *P* values less than 0.05 were considered to indicate statistical significance.

### Data availability

Single-cell RNA-seq data have been deposited in the DNA Data Bank of Japan under accession number DRA011982.

## Results

### Mitochondrial distribution and membrane potential in oocytes produced by IVG and IVM

**Figure 1:**
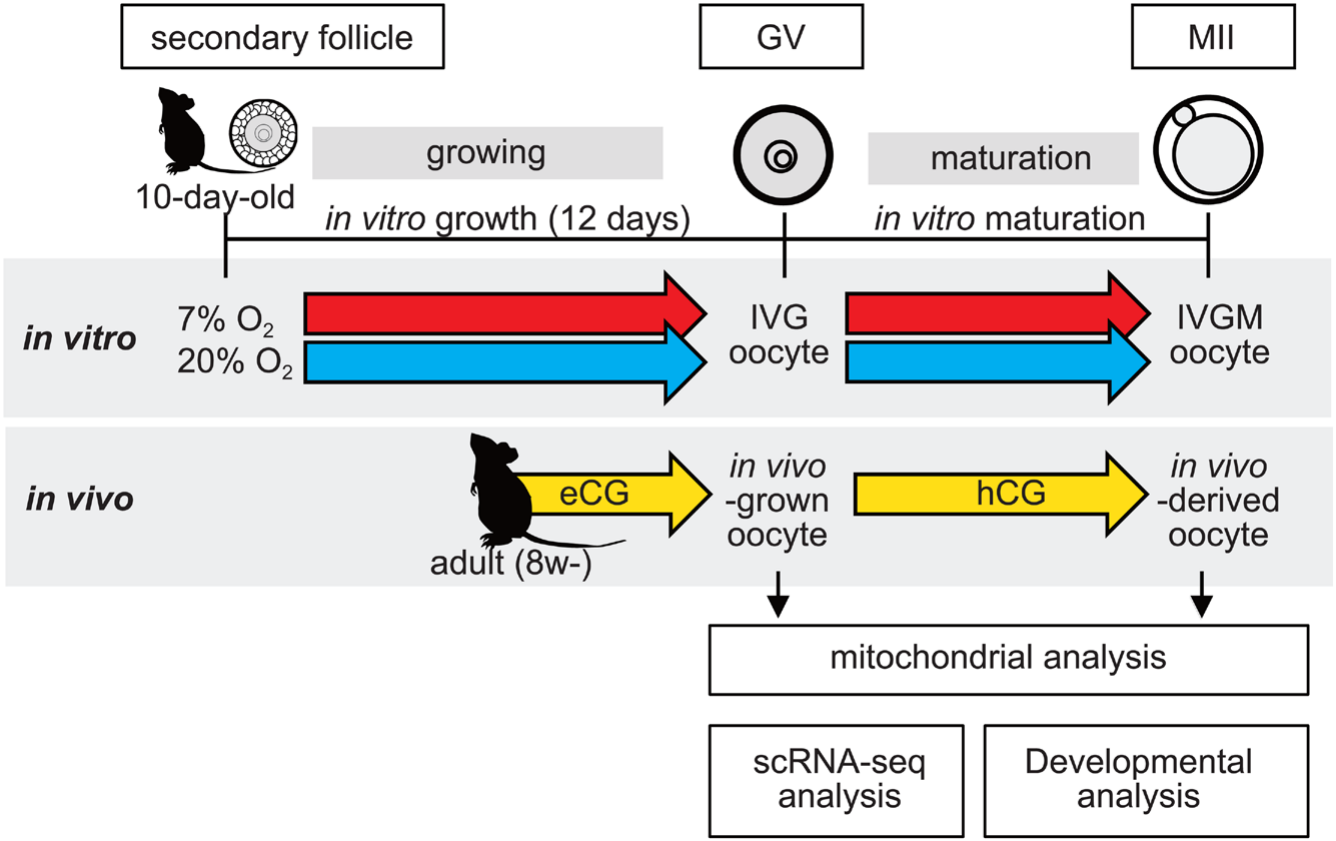
A schematic diagram of experimental design.

**Figure 2:**
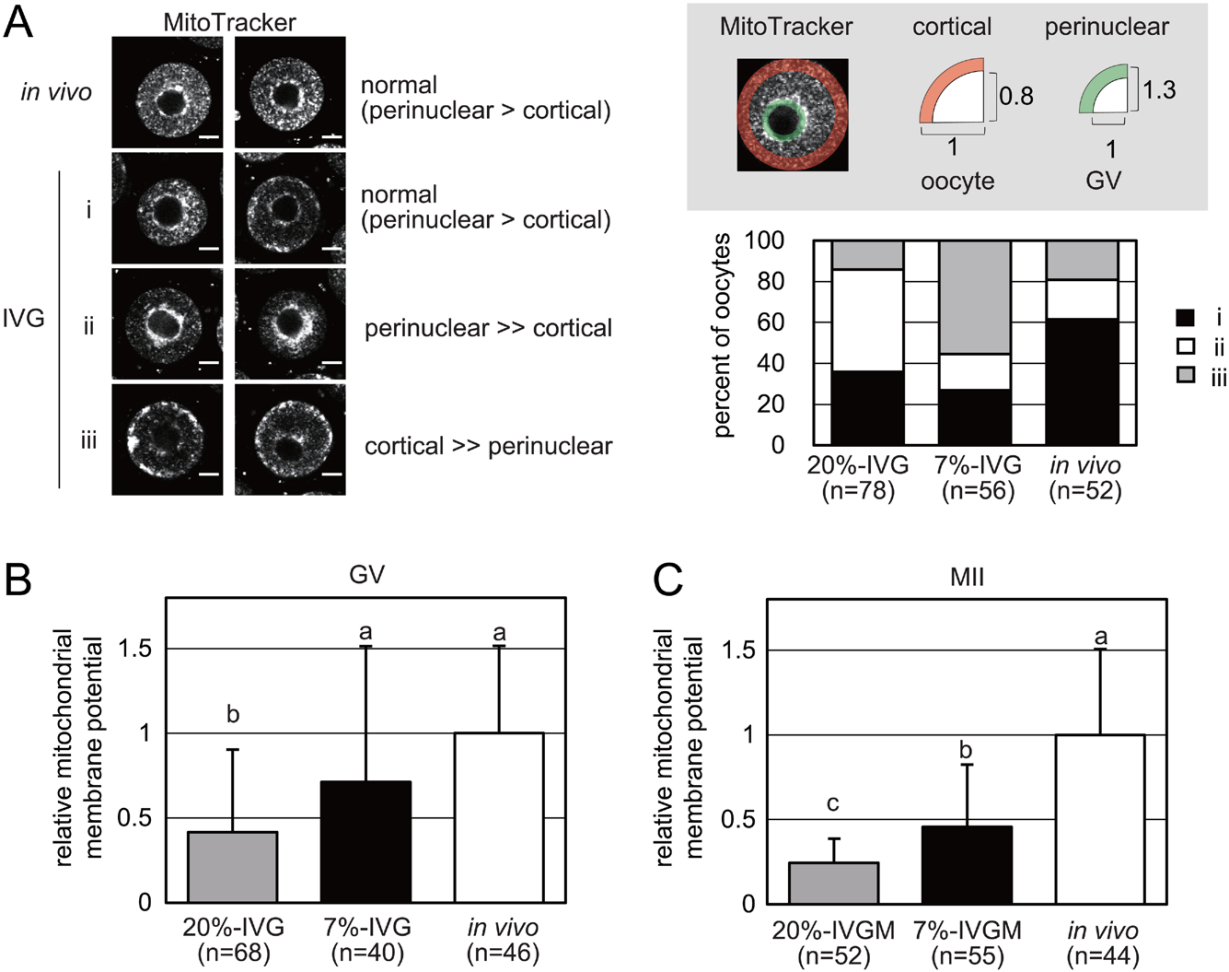
Mitochondrial distribution and membrane potential in *in vivo*-derived oocytes and *in vitro* grown (IVG) and matured (IVGM) oocytes. (A) Representative images of *in vivo*-derived oocyte and IVG oocytes at the germinal vesicle (GV) stages; (i) perinuclear intensity > cortical intensity (black bar, normal); (ii) perinuclear intensity >> cortical intensity (white bars, abnormal); (iii) cortical intensity >> perinuclear intensity (gray bars, abnormal). Scale bars indicate 20 µm. (B and C) Mitochondrial membrane potential of oocytes at the GV (B) and MII (C) stages. Relative values of the ratio of red fluorescence (J-aggregate)/green fluorescence (monomer) in *in vivo*-derived oocytes (white bar) are shown as gray and black bars for 20%-IVG/IVGM and 7%-IVG/IVGM oocytes, respectively. Error bars indicate s.d. Different letters represent significant difference (Tukey–Kramer test, *P*  < 0.05).

We found that mouse oocytes produced via IVG and IVM under 20% O_2_ condition, hereafter referred to as 20%-IVG and 20%-IVGM oocytes (Fig. 1), respectively, were less capable of supporting development after fertilization than *in vivo*-derived oocytes. In contrast, oocytes produced by IVG and IVM under 7% O_2_ condition, hereafter referred to as 7%-IVG and 7%-IVGM oocytes (Fig. 1), respectively, developed to blastocysts after fertilization at a higher frequency, although not high as the *in vivo*-derived oocytes (Supplementary Tables 1 and 2). We hypothesized that this poor competency of IVGM oocytes is due to mitochondrial damage. To test this hypothesis, we first analyzed the mitochondrial distribution and membrane potential in IVG/IVGM oocytes. In *in vivo*-grown oocytes, mitochondria were distributed throughout the cytoplasm and were highly localized around the GV. However, abnormal mitochondrial distribution was observed in 20%-IVG and 7%-IVG oocytes at high frequencies. Mitochondria aggregated extremely around GV or cortical region (Fig. 2A). The relative levels of J-aggregate/monomer at the GV stage were significantly lower in 20%-IVG oocytes (0.4 ± 0.49, *n* = 68, *P*  < 0.05) compared to that in 7%-IVG oocytes (0.7 ± 0.80, *n* = 40) and *in vivo*-grown oocytes (1.0 ± 0.52, *n* = 46) (Fig. 2B). At the MII stage, relative levels of J-aggregate/monomer were also significantly lower in 20%-IVGM (0.2 ± 0.14, *n* = 52, *P*  < 0.05) and 7%-IVGM (0.5 ± 0.37, *n* = 55, *P*  < 0.05) oocytes than that *in vivo*-derived oocytes (1.0 ± 0.51, *n* = 44) (Fig. 2C). Thus, mitochondrial membrane potential was remarkably lower in IVG/IVGM oocytes than that *in vivo*-derived oocytes. However, reduction in mitochondrial membrane potential of IVG/IVGM oocytes was significantly relieved by culturing in 7% O_2_ (*P*  < 0.05).

### Mitochondrial quantity and quality in oocytes produced by IVG and IVM

**Figure 3:**
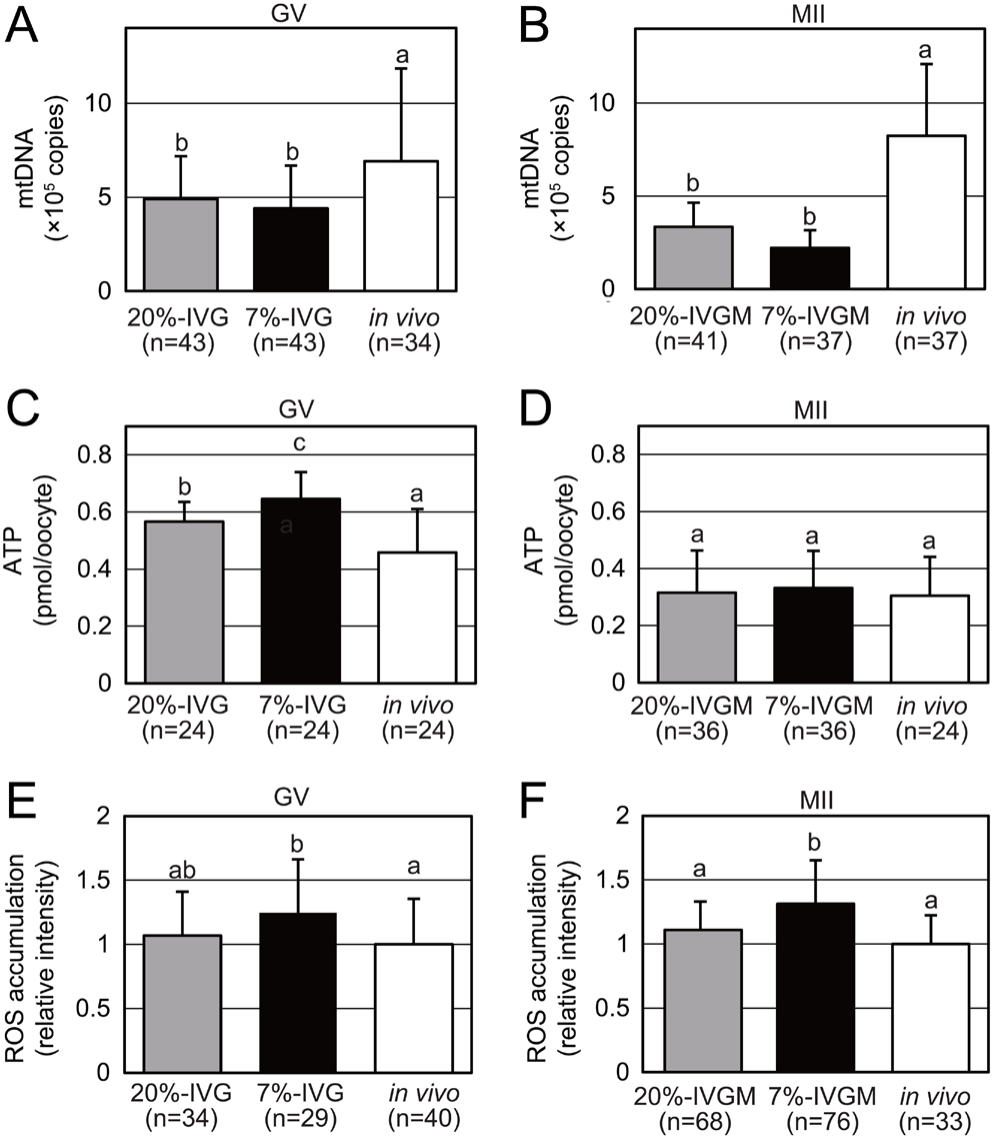
Mitochondrial quantity, ATP content, and ROS accumulation in *in vivo*-derived and *in vitro* grown (IVG) and matured (IVGM) oocytes. (A and B) Quantification of mtDNA copy numbers per oocyte in *in vivo*-derived, 20%-IVG/IVGM, and 7%-IVG/IVGM oocytes at the GV (A) and the MII (B) stages. (C and D) Quantification of ATP content per oocyte in *in vivo*-derived, 20%-IVG/IVGM, and 7%-IVG/IVGM oocytes at the GV (C) and the MII (D) stages. (E and F) ROS accumulation in 20%-IVG/IVGM and 7%-IVG/IVGM oocytes in terms of relative green fluorescence intensity (CellROX Green) of *in vivo*-derived oocytes at the GV (E) and the MII (F) stages. Error bars indicate s.d. Different letters represent significant difference (Tukey–Kramer test, *P*  < 0.05).

To determine whether culture conditions affect mitochondrial quantity and quality, we assessed the mtDNA copy number and ATP content in individual oocytes. At the GV stage, the mtDNA copy number was significantly reduced in 20%-IVG (4.9 × 10^5^ copies, *n* = 43, *P*  < 0.05) and 7%-IVG (4.4 × 10^5^ copies, *n* = 43, *P*  < 0.05) oocytes compared to that in *in vivo*-grown oocytes (6.9 × 10^5^ copies, *n* = 34) (Fig. 3A). These differences became more critical at the MII stage as the mtDNA copy number increased in *in vivo*-derived oocytes (8.2 × 10^5^ copies, *n* = 37) compared to that in the GV stage. In contrast, the mtDNA copy number decreased in both 20%-IVGM and 7%-IVGM oocytes at the MII stage compared with that at the GV stage (20%-IVG, 4.9 × 10^5^ copies, *n* = 43; 7%-IVG, 4.4 × 10^5^ copies, *n* = 43). There was no significant difference in the mtDNA copy number between 20%-IVGM and 7%-IVGM oocytes (Fig. 3B). Consequently, mitochondrial quantity was significantly lower in both 20%-IVGM (*P*  < 0.05) and 7%-IVGM (*P*  < 0.05) oocytes than that *in vivo*-derived oocytes. Unexpectedly, the ATP content in 20%-IVG (0.57 ± 0.070 pmol, *n* = 24, *P*  < 0.05) and 7%-IVG (0.65 ± 0.095 pmol, *n* = 24, *P*  < 0.05) oocytes was greater than that *in vivo*-grown oocytes (0.46 ± 0.153 pmol, *n* = 24) at the GV stage (Fig. 3C). Unlike the GV stage, there was no difference in ATP content among *in vivo*-derived (0.30 ± 0.137 pmol, *n* = 24), 20%-IVGM (0.32 ± 0.147 pmol, *n* = 36), and 7%-IVGM (0.33 ± 0.130 pmol, *n* = 36) oocytes at the MII stage (Fig. 3D). The ROS levels, indicated by fluorescent intensity of the probe, were significantly increased in 7%-IVG oocytes (1.2 ± 0.42, *n* = 29, *P*  < 0.05) and slightly increased in 20%-IVG oocytes (1.1 ± 0.34, *n* = 34) compared to that in *in vivo*-grown GV oocytes (1.0 ± 0.36, *n* = 40) (Fig. 3E). A similar tendency was observed in oocytes at the MII stage (Fig. 3F), suggesting that observed differences in ROS levels do not have an impact on developmental competence.

To summarize, IVG/IVGM oocytes exhibited reduced mitochondrial membrane potential although the culture condition with 7% O_2_ rescues this abnormality to some extent. Additionally, abnormal mitochondrial distribution and diminished mitochondrial quantity were common abnormality in 20%-IVG/IVGM and 7%-IVG/IVGM oocytes.

### Comprehensive analysis of transcriptome of oocytes produced by IVG

**Figure 4:**
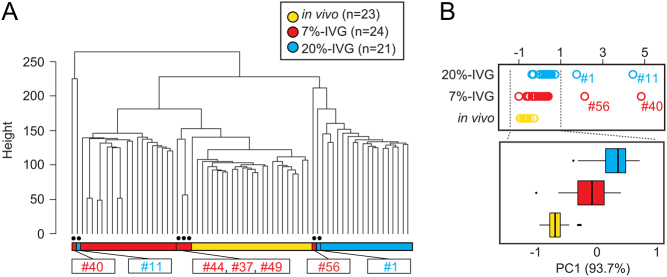
Gene expression profiles of *in vivo*-grown and *in vitro* grown (IVG) oocytes. (A) Hierarchical clustering analysis for all expressed genes (24,335 genes) identified by scRNA-seq of *in vivo*-grown oocytes (*n* = 23; yellow), 20%-IVG oocytes (*n* = 21; blue), and 7%-IVG (*n* = 24; red) oocytes. Among IVG oocytes, one 20%-IVG oocyte (#8) and three 7%-IVG oocytes (#44, #37, and #49) were included in the same cluster as *in vivo*-grown oocytes, while one 20%-IVG oocyte (#11) and one 7%-IVG oocyte (#40) differed from all other oocytes. (B) PCA of scRNA-seq data of *in vivo*-grown oocytes (*n* = 23; yellow), 20%-IVG oocytes (*n* = 21; blue), and 7%-IVG (*n* = 24; red) oocytes. Plots of two 20%-IVG oocytes (#1 and #11) and two 7%-IVG oocytes (#56 and #40) were separated from others on the PC1 axis.

To elucidate the cause of poor competency of IVGM oocytes and lower mitochondrial content and membrane potential in IVG/IVGM oocytes, we performed scRNA-seq analysis in 20%-IVG, 7%-IVG, and *in vivo*-grown oocytes at the GV stage. A total of 24,335 genes were detected in 20%-IVG (*n* = 21), 7%-IVG (*n* = 24), and *in vivo*-grown (*n* = 23) oocytes. Hierarchical clustering analysis revealed differences among the three groups, and transcriptome profiles of 20%-IVG oocytes were distinct from those of 7%-IVG and *in vivo*-grown oocytes (Fig. 4A). Interestingly, three 7%-IVG oocytes (#44, #37, and #49) were included in the *in vivo*-grown oocyte cluster. PCA showed that the distance among 20%-IVG, 7%-IVG, and *in vivo*-grown oocytes was not widely separated but shifted to the left side on the PC1 axis (93.7%) in increasing order of oocyte competency (Fig. 4B). Two oocytes from each of 20%-IVG (#1 and #11) and 7%-IVG (#40 and #56) oocyte groups were plotted on the right side of the clusters. These results suggest that the differences shown by hierarchical clustering analysis and PCA reflect the differences in oocyte competency. Furthermore, GO analysis of the 1450 genes that contributed highly to the PC1 axis variation (more than 2 s.d.) showed enrichment of GO terms such as 'negative regulation of apoptotic process' (Supplementary Fig. 1). This suggests that the variance of the PC1 axis may be associated with the mitochondrial abnormalities in the IVG oocytes described above.

Then, we investigated mitochondria-associated genes (Supplementary Fig. 2), transcription factor A, mitochondria (*Tfam*), that are involved in mitochondria biogenesis was significantly downregulated in 20%-IVG oocytes (padj < 0.05) but not in 7%-IVG oocytes compared with *in vivo*-grown oocytes. The expression of nuclear respiratory factor 1 (*Nrf1*), a *Tfam* upstream regulator, did not significantly vary among the groups, while that of dynamin 1-like (*Dnm1l*, also known as *Drp1*) and mitofusin 1 (*Mfn1*), which are essential for mitochondrial fission and fusion, respectively (Hall *et al.* 2014, Novin *et al.* 2015), were significantly downregulated in 7%-IVG oocytes compared to *in vivo*-grown oocytes (padj < 0.05). On the other hand, several mitochondria-associated genes coded by mtDNA that are involved in oxidative phosphorylation were upregulated in 20%-IVG oocytes (padj < 0.05) but not in 7%-IVG oocytes. The expression levels of well-known mitochondria-associated genes did not delineate the network.

### Profile of IVG oocytes

**Figure 5:**
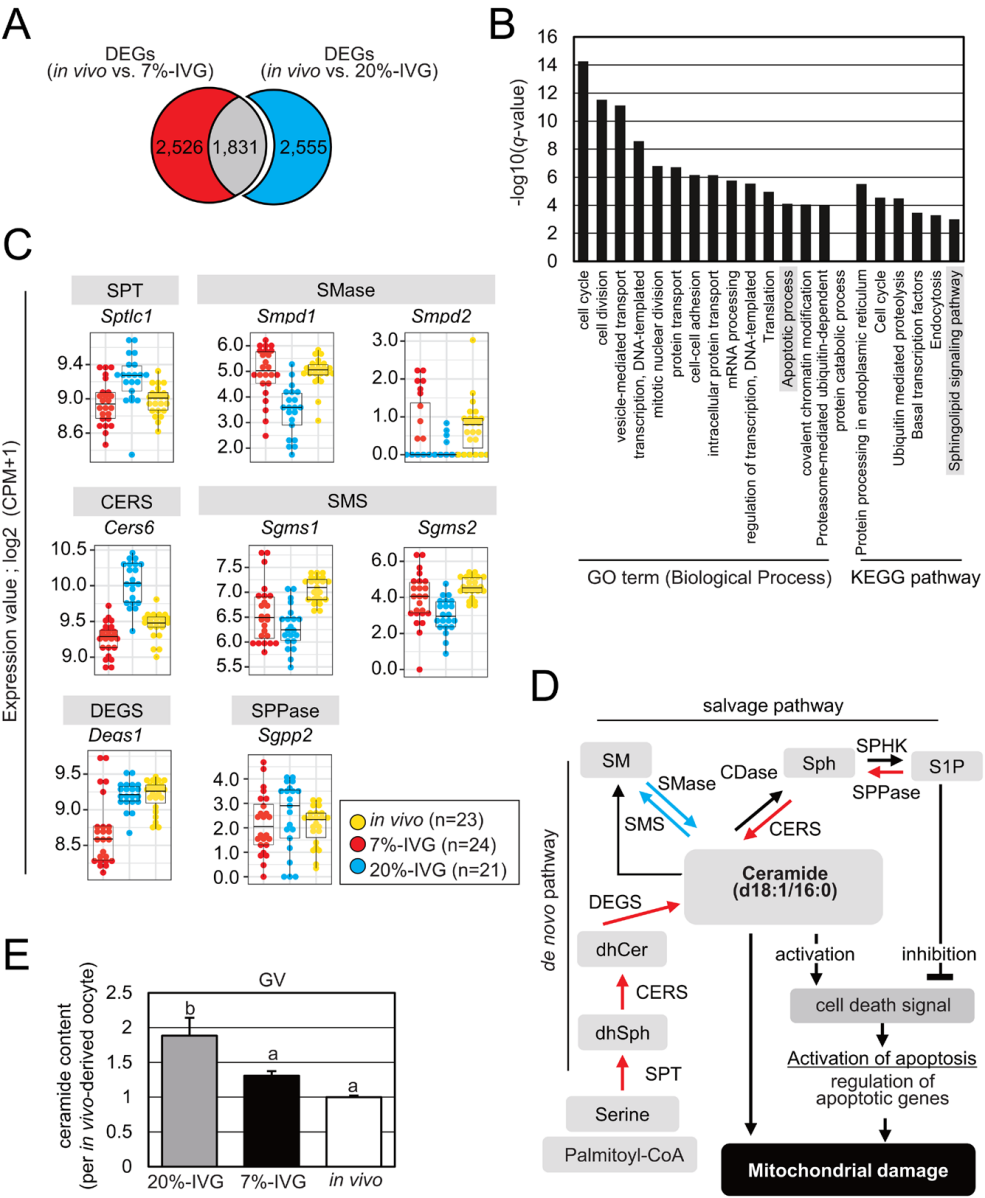
Identification of abnormal pathway specific to *in vitro* grown (IVG) oocytes under atmospheric O_2_ culture condition. (A) Venn diagram of 4357 differentially expressed genes (DEGs; red and gray; padj < 0.05) between 7%-IVG and *in vivo*-grown oocytes and 4386 DEGs (blue and gray; padj < 0.05) between 20%-IVG and *in vivo*-grown oocytes. A total of 2555 DEGs (blue) were specific to 20%-IVG oocytes. (B) Enrichment analysis of the 20%-IVG oocyte-specific 2555 DEGs (*q*-value < 0.05). Vertical axis indicates −log_10_(*q*-value value). (C) Expression of sphingolipid metabolic pathway genes included in the 2555 20%-IVG oocyte-specific DEGs. The dots indicate expression in *in vivo*-grown (yellow), 20%-IVG (blue), and 7%-IVG (red) oocytes. Vertical axis indicates log_2_(CPM+1). SPT, serine palmitoyltransferase; SMase, sphingomyelinase; CERS, ceramide synthase; SMS, Sphingomyelin synthase; DEGS, dihydroceramide desaturase; SPPase, sphingosine-1-phosphate phosphatases. (D) A schematic diagram of the sphingolipid metabolic pathway consisting of the* de novo* and the salvage pathways. Red and blue arrows indicate significantly upregulated and downregulated gene expression, respectively, in 20%-IVG oocytes. dhSph, dihydrosphingosine; dhCer, dihydroceramide; Sph, sphingosine; S1P, sphingosine-1-phosphate; CDase, ceramidase; SPHK, sphingosine kinase. (E) C16-ceramide content in *in vivo*-grown, 20%-IVG, and 7%-IVG oocytes. Ceramide quantity in 20%-IVG and 7%-IVG oocytes relative to that in *in vivo*-grown oocyte. Error bars indicate s.d. Different letters represent significant difference (Tukey–Kramer test, *P*  < 0.05).

To explore molecular mechanisms affecting mitochondrial function and oocyte competency, we analyzed DEGs in IVG oocytes. As shown in Fig. 5A, we identified 4386 and 4357 DEGs between* in vivo*-grown oocytes and 20%-IVG and 7%-IVG oocytes, respectively (Supplementary Table 3). GO analysis of 20%-IVG oocyte-specific DEGs (2,555 genes) showed enrichment of 'cell cycle', 'cell division', etc. (Fig. 5B). Furthermore, genes involved in 'apoptotic process' were included in 20%-IVG oocyte-specific DEGs, suggesting mitochondrial dysfunction (Supplementary Table 4). KEGG pathway analysis of 20%-IVG oocyte-specific DEGs revealed enrichment in 'protein processing in the endoplasmic reticulum', 'cell cycle', and 'sphingolipid signaling pathway', among others. Notably, one sphingolipid synthetic pathway function is to act as an upstream regulator of apoptosis, that is, mitochondrial dysfunction. Ceramide, a major sphingolipid, acts as a proapoptotic signaling molecule and reduces mitochondrial membrane potential, whereas sphingosine 1-phosphate (S1P) protects mitochondria from apoptosis (Cartier & Hla 2019). In 20%-IVG oocytes, expression of the genes encoding enzymes essential for* de novo* synthesis of ceramide from serine and palmitate, namely, serine palmitoyltransferase, long chain base subunit 1 (*Sptlc1*), ceramide synthase 6 (*Cers6*), and delta(4)-desaturase, sphingolipid 1 (*Degs1*) was significantly upregulated (Fig. 5C and D). Furthermore, significant upregulation of sphingosine-1-phosphate phosphatase 2 (*Sgpp2*) and *Cers6* and significant downregulation of sphingomyelin synthase 1 (*Sgms1*) and 2 (*Sgms2*) suggest the facilitation of ceramide production from S1P and suppressed conversion of ceramide to sphingomyelin in 20%-IVG oocytes. Overall, evidence supports the hypothesis that an excess of ceramide is produced and accumulated in 20%-IVG oocytes. To validate this hypothesis, we quantitated ceramide. Of the several ceramide species, only ceramide (d18:1/16:0) could be quantified in oocytes. We found higher ceramide content in 20%-IVG oocytes (1.8 ± 0.26) than *in vivo*-grown (1.0 ± 0.02) and 7%-IVG oocytes (1.3 ± 0.06, *P*  < 0.05; Fig. 5E). Although further study is required to demonstrate that ceramide synthesis is actually increased in 20%-IVG oocytes, excess ceramide level contained in 20%-IVG oocytes may have deleterious effects on oocyte competency.

**Figure 6:**
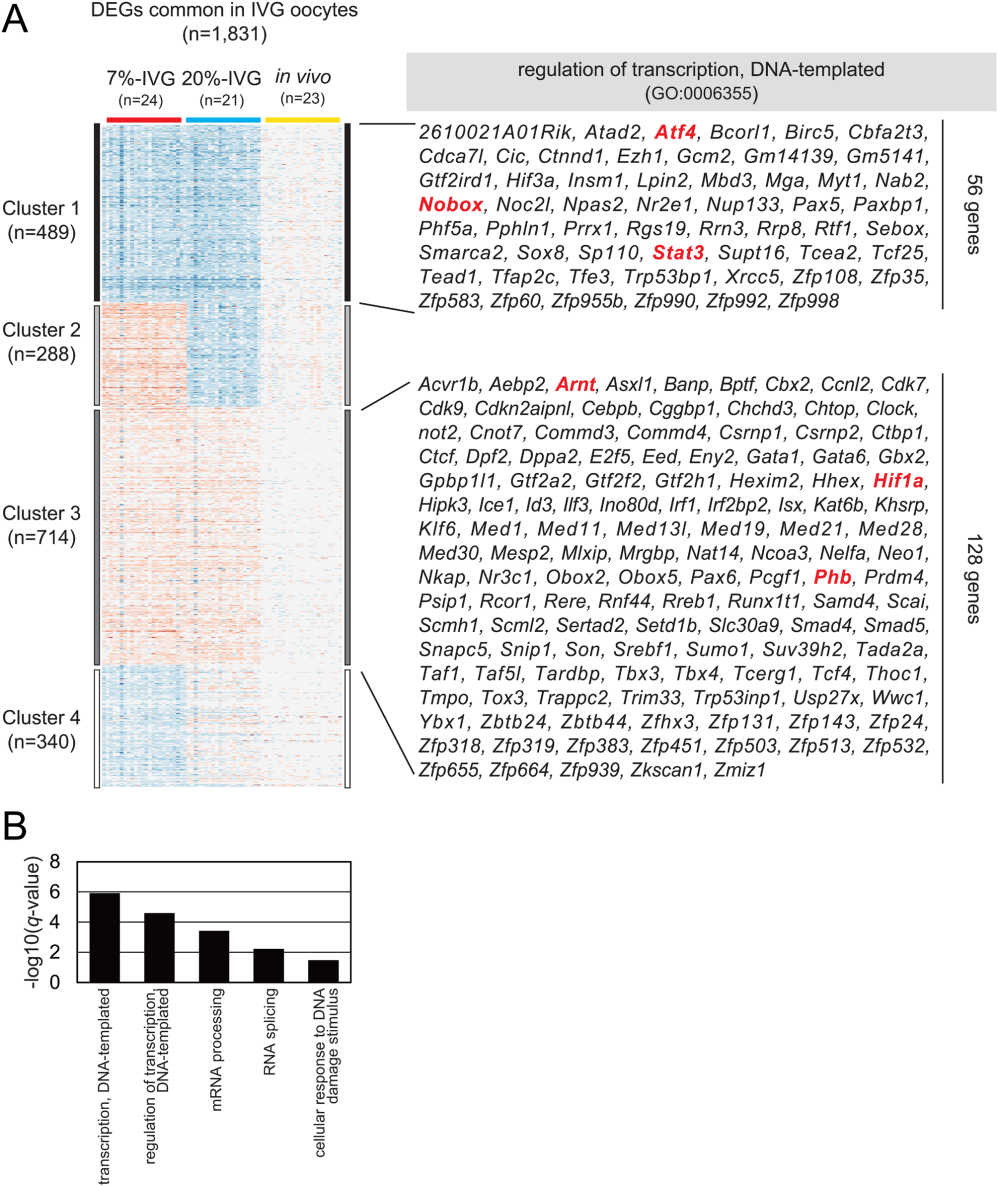
K-means clustering of common differentially expressed genes (DEGs) in *in vitro* grown (IVG) oocytes cultured under different O_2_ concentrations. (A) K-means clustering (K = 4) of 1831 DEGs that were common between 20%-IVG and 7%-IVG oocytes. Cluster 1 (489 genes) and cluster 3 (714 genes) indicate significantly downregulated and upregulated genes, respectively, in 20%-IVG and 7%-IVG oocytes. The list indicates genes annotated as 'regulation of transcription and DNA-templated' in cluster 1 (56 genes) and cluster 3 (128 genes). (B) Enrichment analysis of 1203 DEGs in cluster 1 and cluster 3 (*q*-value < 0.05).

Next, we focused on the common 1831 DEGs (Fig. 5A) in 20%-IVG and 7%-IVG oocytes. K-means clustering (K was defined as 4) was used to identify downregulated (489) and upregulated (714) genes (Fig. 6A) in IVG oocytes. As the GO analysis for 1203 genes included in clusters 1 and 3 revealed that GO term 'transcription, DNA-templated' was enriched as a most representative term (Fig. 6B), we focused on the 184 genes annotated to 'transcription, DNA-templated'. Regardless of the O_2_ concentration in culture, hypoxia-inducible factor 1 alpha (*Hif1a*) and aryl hydrocarbon receptor nuclear translocator (*Arnt/Hif1b*) were upregulated and included in cluster 3. Similarly, *Phb* encoding prohibitin that is localized in the mitochondria was also included in cluster 3. Furthermore, NOBOX oogenesis homeobox (*Nobox*) and signal transducer and activator of transcription 3 (*Stat3*) were downregulated and included in cluster 1. STAT3 has pleiotropic roles and is also known to regulate mitochondrial gene transcription and mitochondrial function (Carbognin *et al.* 2016). Although common DEGs in IVG oocytes did not delineate pathways, the abnormal expression of transcription factors could explain mitochondrial dysfunction and/or poor developmental competency in IVG oocytes.

**Figure 7:**
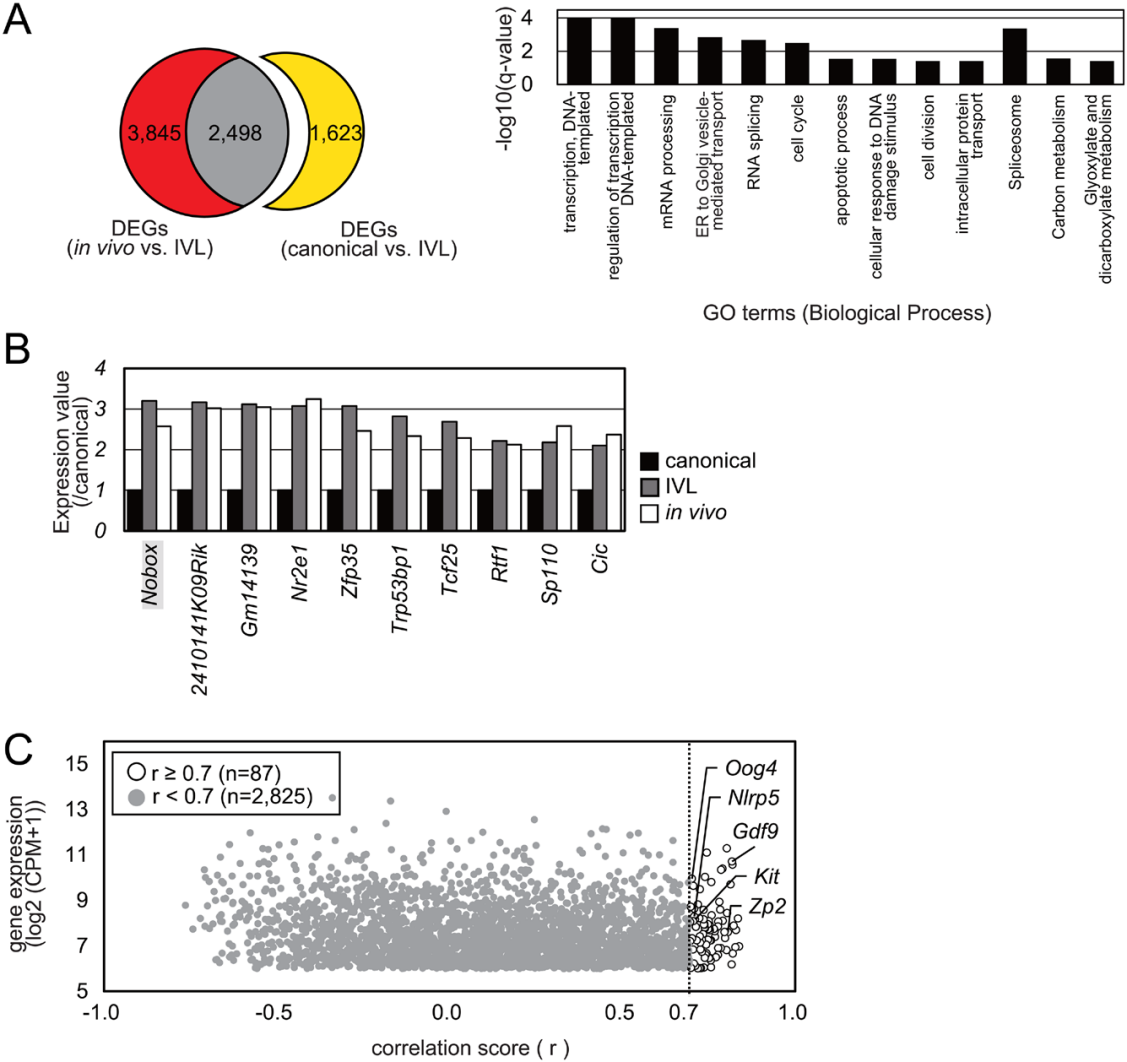
Identification of candidate genes responsible for close clustering of *in vivo*-grown and *in vitro* grown (IVG) oocytes. (A) Venn diagram of 6343 DEGs (red and gray; padj < 0.05) between *in vivo*-grown oocyte-like IVG (IVL) oocytes and *in vivo*-grown oocytes and 4121 DEGs (yellow and gray; padj < 0.05) between canonical 7%-IVG oocytes and IVL oocytes. The expression levels of 1623 genes (yellow) in IVL oocytes were restored to those in *in vivo*-grown oocytes. GO analysis of the 1623 DEGs showed enrichment of GO term 'regulation of transcription and DNA-templated'. Horizontal axis indicates −log_10_(*q*-value). (B) Gene expression levels in IVL (gray) and *in vivo*-grown (white) oocytes relative to that in canonical-7%-IVG oocytes (black). (C) Correlation between the expression of *Nobox* and other genes [log_2_(CPM+1)≥6]. White and gray dots indicate a correlation score ≥ 0.7 and < 0.7, respectively.

Finally, we focused on gene expression profiles of three 7%-IVG oocytes (#44, #37, and #49, Fig. 4A) that were *in vivo*-grown oocyte-like IVG oocytes and named IVL oocytes. There were 4121 DEGs between canonical 7%-IVG oocytes (excluding #40, #56, #44, #37, and #49) and IVL oocytes (Fig. 7A). Of these, 2498 genes were the same as those differentially expressed between IVL oocytes and *in vivo*-grown oocytes, suggesting that the remaining 1623 DEGs were responsible for the close clustering of IVL and *in vivo*-grown oocytes. The expression of these 1623 genes in the IVL oocytes was similar to that in the *in vivo*-grown oocytes. The 56 genes annotated as 'transcription, DNA-templated' were significantly downregulated in IVG oocytes (Fig. 6A). However, the expression of 10 of the 56 genes in IVL oocytes was restored to the level in *in vivo*-grown oocytes (Fig. 7B). Notably, the expression of *Nobox* was comparable between* in vivo*-grown and IVL oocytes. Correlation analysis showed that the expression level of *Nobox* correlated with expression levels of 87 genes (log_2_(CPM+1)≥6, r ≥ 0.7), including growth differentiation factor 9 (*Gdf9*), zona pellucida glycoprotein 2 (*Zp2*), KIT proto-oncogene receptor tyrosine kinase (*Kit*), NLR family, pyrin domain containing 5 (*Nlrp5*), and oogenesin 4 (*Oog4*) (Fig. 7C and Supplementary Table 5). These results suggest the possibility that oocyte competency, which is acquired during oocyte growth, would be impacted by not only mitochondrial function but also transcription factors such as *Nobox*.

## Discussion

Maternal factors, including proteins, mRNA, and organelles, are directly responsible for successful embryonic development. Determining the cause of reduced developmental competence in IVGM oocytes would help to elucidate the mechanism of functional oocyte production* in vivo* and/or identify factors required for establishing oocyte competency. In the present study, we focused on the recovered developmental competency of IVGM oocytes by reducing O_2_ concentration in the culture from atmospheric (20%) to physiological (7%) levels. We investigated the role of mitochondria in the recovery of oocyte competency and identified the pathway impacting mitochondrial function and oocyte competency by molecular analyses.

First, we found that mitochondria in 20%-IVG and 7%-IVG oocytes were abnormally aggregated in perinuclear and cortical regions, respectively. The cause of this difference in distribution was unclear in this study. Several studies have shown that abnormal mitochondrial distribution in oocytes correlates with the low mitochondrial quality and low meiotic and developmental competencies (Stojkovic *et al.* 2001, Brevini *et al.* 2005, Nagai *et al.* 2006). However, IVGM oocytes reached the MII stage at a high frequency regardless of the O_2_ environment. In contrast, the mitochondrial membrane potential of 20%-IVG/IVGM oocytes was significantly reduced compared with that in 7%-IVG/IVGM and *in vivo*-derived oocytes, suggesting that attenuation of mitochondrial abnormalities could be one of the causes responsible for recovered developmental competence of 7%-IVGM oocytes compared with those of 20%-IVGM oocytes. scRNA-seq analysis identified abnormalities in the expression of genes associated with apoptosis and sphingolipid signaling pathway, which includes ceramide metabolism, in 20%-IVG oocytes. Ceramide not only directly induces mitochondrial damage but plays a role as a lipid second messenger to mediate activation of apoptosis-associated genes and thereby indirectly induces mitochondrial damage (Gudz *et al.* 1997, Haimovitz-Friedman *et al.* 1997). In 20%-IVG oocytes, the expression of gene sets associated with ceramide synthesis was significantly upregulated. *Cers6*, which was markedly overexpressed in 20%-IVG oocytes, is required for the synthesis of C16-fatty acid-containing ceramide. The most compelling evidence supporting abnormal ceramide metabolism was the significantly higher C16-fatty acid ceramide (d18:1/16:0) content in 20%-IVG oocytes than that in 7%-IVG and *in vivo*-grown oocytes. Itami *et al.* showed that adding ceramide or palmitate, a ceramide source, to the IVM medium detrimentally affects meiotic competency and mitochondrial function in porcine oocytes (Itami *et al.* 2018). Moreover, ceramide content was reportedly higher in oocytes collected from aged mice than that in oocytes from young mice (Perez *et al.* 2005). Taken together, we concluded that ceramide metabolism is affected by O_2_ concentration during IVG, and excess ceramide accumulation in 20%-IVG oocytes leads to mitochondrial dysfunction and impaired developmental ability. Indeed, follicular O_2_ concentration reportedly reduces with follicular growth in the ovaries of humans, cows, and pigs unlike in *in vitro* constitutive environment (Fischer *et al.* 1992, de Castro *et al.* 2008). Thus, reduction in follicular O_2_ concentration possibly inhibits excess ceramide production in oocytes, which in turn ensures mitochondrial integrity in *in vivo-*grown oocytes.

Next, we found that mtDNA copy number was reduced in both IVGM oocytes. Additionally, the IVG oocytes contained higher ATP content than* in vivo*-grown oocytes at the GV stage although a significant reduction in mitochondrial membrane potential in IVG oocytes compared to that in *in vivo*-derived oocytes. A previous study showed that mitochondrial cristae change from irregular to transverse and mitochondrial matrix becomes more denser with progression from non-growing to fully grown stage (Sathananthan & Trounson 2000). This suggests that mitochondrial ATP production activity is acquired by the growth phase transition in oocytes. Genetic and biochemical analyses showed that glycolysis is not active in oocytes but the expression of genes associated with oxidative phosphorylation is increased after entering the growth phase (Shimamoto *et al.* 2019). In contrast, despite the oocyte-specific deletion of pyruvate dehydrogenase E1 alpha1 (*Pdha1*), growth and ATP accumulation were observed in GV oocytes (Johnson *et al.* 2007), indicating that ATP in oocytes is either completely supplied by follicle cells, as in the case of *Pdha1* deletion, or partially supplemented by these cells during oocyte growth; nevertheless, ATP production in oocytes during growth remains to be elucidated. On the other hand, oocytes are known to produce ATP from pyruvate and consume ATP during meiotic maturation (Downs 1995, Yu *et al.* 2010, Dalton *et al.* 2014). In the present study, higher ATP content in IVG oocytes was found to be decreased to the same level as that in *in vivo*-derived oocytes after IVM. This implies that the rate of ATP consumption was more than that of ATP production in IVG oocytes during IVM, which may be attributed to mitochondrial dysfunction. The effects of reduced mitochondrial membrane potential and quantity are evident after the meiotic resumption due to the reduction in excess ATP levels in IVG oocytes which is presumably associated with their subsequent developmental fate. The reason and mechanism for greater ATP accumulation in IVG oocytes than that in in* vivo*-grown oocytes remain unclear; however, our findings suggest that currently employed IVG culture conditions would cause metabolic alterations in oocytes and/or the surrounding follicle cells. Additionally, the causes of mitochondrial damage in IVG oocytes other than excess ceramide accumulation have not been identified. Recent studies have shown that STAT3 is localized in mitochondria and contributes to activating ATP production and inhibiting ROS production in somatic cells (Carbognin *et al.* 2016). *Stat3* is also involved in transcription and chromatin dynamics in oocytes and early embryos. Considering the fact that STAT3, produced in the non-growing oocytes, persists even in conditionally *Stat3*-deleted fully grown oocytes by *Gdf9*-Cre (Haraguchi *et al.* 2020), understanding of the maternal STAT3 function is limited (Ou-Yang *et al.* 2021). However, *Stat3* that was downregulated in both IVG oocytes and might be responsible for mitochondrial dysfunction in IVG oocytes.

Finally, we used a data mining strategy to identify candidate genes impacting oocyte competency. scRNA-seq analysis showed that four IVG oocytes were classified into the same cluster as* in vivo*-grown oocytes. Among these, we focused on three 7%-IVG oocytes that were IVL oocytes, based on the hypothesis that these oocytes exhibit full competency similar to *in vivo*-grown oocytes. Then, we identified ten genes that were downregulated in IVG oocytes and found that the expression of genes annotated to 'transcription, DNA-templated' was recovered in IVL oocytes. Among these ten genes, only *Nobox* is known to be essential for oocyte development. In *Nobox*-deleted female mice, oogenesis ceased before oocyte growth with concomitant abolishment of *Gdf9* expression (Rajkovic *et al.* 2004). NOBOX binds to the promoter of *Gdf9* and *Pou5f1* (Choi & Rajkovic 2006). *Gdf9* is expressed in oocytes and is essential for follicle growth (Carabatsos *et al.* 1998). In the present study, expression levels of 87 genes including *Gdf9* clearly correlated with that of *Nobox*. *Kit* and *Nlrp5*, whose expression levels were correlated with that of *Nobox*, are also essential maternal factors (Nishijima *et al.* 2014, Monk *et al.* 2017). Till date, *Nobox* function has been studied mainly before oocyte growth; however, *Nobox* may control the expression of gene networks throughout oogenesis and thereby regulate the acquisition of oocyte competency.

In conclusion, we showed that IVG oocytes exhibited mitochondrial dysfunction, and this may be partially attributed to excess ceramide accumulation resulting from abnormal metabolism. Our findings indicate that ceramide metabolism is regulated by O_2_ concentration during oocyte growth. The expression levels of transcription factors were altered in IVG oocytes but recovered in IVL oocytes. Further studies on cell metabolism in IVG oocytes/follicles and functional analysis of *Nobox* may open new avenues for understanding the mechanisms responsible for acquiring oocyte competency.

## Supplementary Material

Supplementary Materials

Figure S1 Transcriptome analysis of in vivo-grown and in vitro grown (IVG) oocytes Orange bars indicate the number of genes that substantially contributed to the PC1 axis variation (more than 2 S.D.). Gray bars indicate the number of genes that contributed less to the PC1 axis variation (within 2 S.D.). GO analysis of the 1,450 genes that contributed highly to PC1 axis variation. Several of these corresponded to the results of the GO analysis of 20%-IVG oocyte-specific DEGs (Figure 5B).

Figure S2 Expression of mitochondria-associated genes Expression of mitochondria-associated genes in in vivo-grown oocytes (yellow), 20%-IVG oocytes (blue), and 7%-IVG oocytes (red). Vertical axis indicates log₂ (CPM+1). Asterisks indicate significant difference (padj<0.05).

Table S1 Growth and maturation of oocytes in vitro

Table S2 Developmental ability of in vitro grown (IVG) oocytes after in vitro fertilization

Table S3 Differentially expressed genes in in vitro grown (IVG) oocytes

Table S4 Expression of the genes annotated as “apoptotic process” in Figure 4B

Table S5 Genes highly correlated with Nobox

## Declaration of interest

The authors declare that there is no conflict of interest that could be perceived as prejudicing the impartiality of the research reported.

## Funding

This work was supported in part by KAKENHI (18H05547 to Y O) and the Sasakawa Scientific Research Grant from the Japan Science Society http://dx.doi.org/10.13039/501100007807 (2020-4090 to T T).

## Author contribution statement

This study was designed by Y O. All experiments were conducted by T T and T F. All data were analyzed by T T. The manuscript was written by Y O and T T.
