# Supplementary material for: Effect of in vitro growth on mouse oocyte competency, mitochondria and transcriptome
Source: Reproduction. 2021 Aug 12;162(4):307–18. doi: 10.1530/REP-21-0209 (PMC8494378; doi:10.1530/REP-21-0209)
Supplement: Supplementary Materials [file supplementary_material.pdf]

Supplementary Materials and Methods

Culture

All culture experiments for oocyte and embryo production were conducted as described previously (Morohaku *et al.* 2017). For IVG, secondary follicles from 10-day-old mice were isolated and then treated with 0.1% (w/v) collagenase type I (Worthington Biochemicals) in L15 medium for 28 min at 37 °C. These secondary follicles were cultured on Transwell-COL insert membrane (Corning) in a 6-well plate with minimum essential medium (MEM) alpha (Gibco) supplemented with 2% (w/v) PVP (Sigma-Aldrich), 5% (v/v) fetal bovine serum (FBS; Sigma-Aldrich), and 0.1 IU/mL follicle stimulating hormone (FSH; MSD) for 12 days at 37 °C under atmospheric (5% CO_2 and 95% air, i.e., 20% O_2) or physiological (5% CO_2, 7% O_2, and 88% N_2) O_2 conditions.

Cumulus–oocyte complexes (COCs), isolated from explants at day 12 of culture, were cultured in MEM alpha supplemented with 5% (v/v) FBS, 0.1 IU/mL FSH, 1.2 IU/mL human chorionic gonadotropin (hCG; ASKA Pharmaceutical), and 4 ng/mL epidermal growth factor (Gibco) for 17 h under the same conditions as those used for IVG.

Epididymal sperms were collected from adult males. Expanded COCs were cultured with capacitated sperms in TYH medium (LSI Medience) under the same conditions as for *in vitro* growth and maturation (IVGM). Normally fertilized oocytes were cultured in KSOM+AA medium (Millipore) for 5 days under the same conditions as IVGM. Oocytes in the metaphase of second meiosis (MII), collected from adult mice as described below, were used as control. *In vivo*-derived MII oocytes were subjected to IVF similar to the IVGM oocytes.
