# Supplementary material for: Effect of in vitro growth on mouse oocyte competency, mitochondria and transcriptome
Source: Reproduction. 2021 Aug 12;162(4):307–18. doi: 10.1530/REP-21-0209 (PMC8494378; doi:10.1530/REP-21-0209)
Supplement: Figure S1 Transcriptome analysis of in vivo-grown and in vitro grown (IVG) oocytes Orange bars indicate the number of genes that substantially contributed to the PC1 axis variation (more than 2 S.D.). Gray bars indicate the number of genes that contributed less to the PC1 axis variation (within 2 S. [file supplementary_figure_1.pdf]

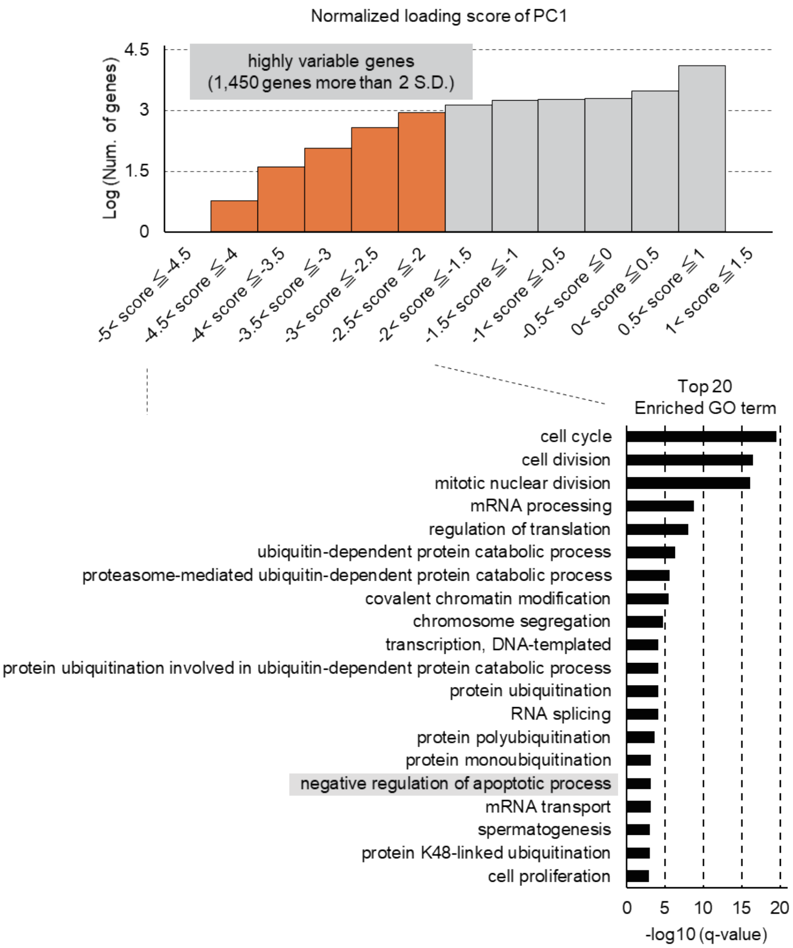

Figure S1 Transcriptome analysis of *in vivo*-grown and *in vitro* grown (IVG) oocytes

Orange bars indicate the number of genes that substantially contributed to the PC1 axis variation (more than 2 S.D.). Gray bars indicate the number of genes that contributed less to the PC1 axis variation (within 2 S.D.). GO analysis of the 1,450 genes that contributed highly to PC1 axis variation. Several of these corresponded to the results of the GO analysis of 20%-IVG oocyte-specific DEGs (Figure 5B).
