# Supplementary material for: Effect of in vitro growth on mouse oocyte competency, mitochondria and transcriptome
Source: Reproduction. 2021 Aug 12;162(4):307–18. doi: 10.1530/REP-21-0209 (PMC8494378; doi:10.1530/REP-21-0209)
Supplement: Figure S2 Expression of mitochondria-associated genes Expression of mitochondria-associated genes in in vivo-grown oocytes (yellow), 20%-IVG oocytes (blue), and 7%-IVG oocytes (red). Vertical axis indicates log₂ (CPM+1). Asterisks indicate significant difference (padj<0.05). [file supplementary_figure_2.pdf]

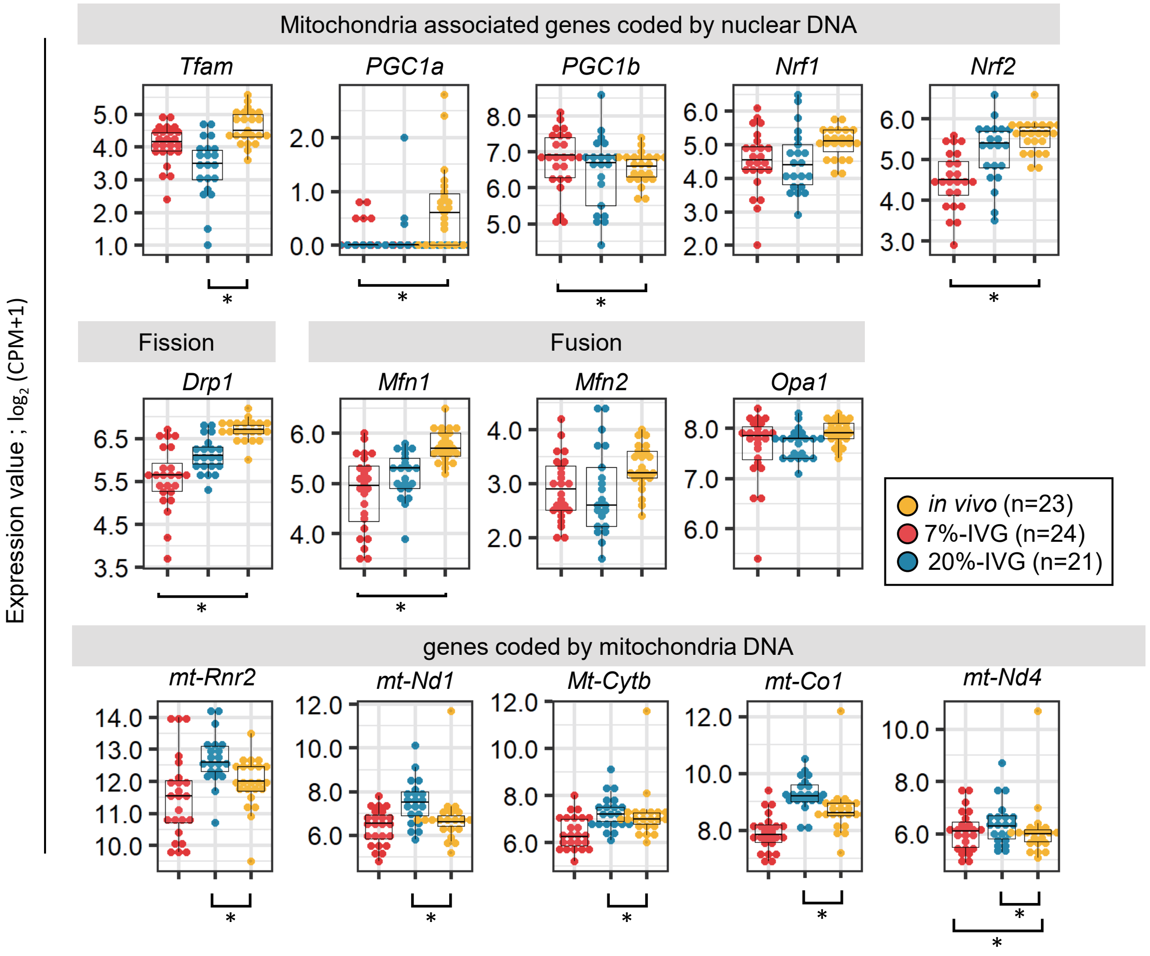

Figure S2 Expression of mitochondria-associated genes

Expression of mitochondria-associated genes in *in vivo*-grown oocytes (yellow), 20%-IVG oocytes (blue), and 7%-IVG oocytes (red). Vertical axis indicates $\log_2$ (CPM+1). Asterisks indicate significant difference (padj<0.05).
